# Breast density and outcome of mammography screening: a cohort study

**DOI:** 10.1038/sj.bjc.6604989

**Published:** 2009-03-17

**Authors:** A H Olsen, K Bihrmann, M-B Jensen, I Vejborg, E Lynge

**Affiliations:** 1 Department of Epidemiology, Institute of Public Health, University of Copenhagen, Øster Farimagsgade 5, København K DK-1014, Denmark; 2 Danish Breast Cancer Cooperative Group, University Hospital Copenhagen, Blegdamsvej 9, Copenhagen Ø DK-2100, Denmark; 3 Centre of Diagnostic Imaging, University Hospital Copenhagen, Blegdamsvej 9, Copenhagen Ø DK-2100, Denmark

**Keywords:** malignant neoplasms, breast, mammography, screening, mortality, breast density

## Abstract

The purpose of this study was to investigate the effect of breast density on breast cancer (BC) mortality in a mammography screening programme. The cohort included 48 052 women participating in mammography screening in Copenhagen, Denmark, where biennial screening is offered to women aged 50–69 years. We collected information for the years 1991–2001 on screening outcome, incident BCs (screen-, interval-, and later detected), and BC deaths. Breast density was dichotomised into fatty (F) and mixed/dense (M/D) breasts. Screening sensitivity was measured as the odds ratio of interval *versus* screen-detected cancer for dense *versus* F breasts. Poisson regression was used to estimate the ratios for BC incidence, case fatality, and mortality between women with M/D and F breasts. For women with M/D breasts, the odds ratio of an interval cancer was 1.62 (95% confidence interval, CI, 1.14–2.30), and the age-adjusted rate ratios were 2.45 (95% CI 2.14–2.81) for BC incidence, 0.60 (95% CI 0.43–0.84) for case fatality, and 1.78 (95% CI 1.17–2.72) for BC mortality. The study shows that BC in women with M/D breasts is more frequent, but on average less severe, than in women with F breasts.

Breast density is a measure of the composition of the breast tissue. Breasts with low density have a high proportion of fatty (F) tissue, whereas breasts with high density have a high proportion of epithelial and connective tissue. Mammography has a lower sensitivity in women with mixed/dense (M/D) breasts than in women with F breasts (Mandelson *et al*, 2000, Ciatto *et al*, 2004; Chiarelli *et al*, 2006), and women with M/D breasts have a higher incidence of breast cancer (BC) than women with F breasts (McCormack and dos Santos Silva, 2006; Boyd *et al*, 2007). We therefore examined whether breast density affects the outcome of mammography screening using data from the organised mammography screening programme in Copenhagen, Denmark, earlier shown to reduce BC mortality in targeted women by 25% and in participating women by 37% (Olsen *et al*, 2005).

We tested the following hypotheses: compared with women with F breasts, (1) the sensitivity of mammography is lower in women with M/D breasts, and (2) women with M/D breasts have a higher BC incidence. Our results were well in accordance with those of earlier studies. We further tested whether: (3) because of the lower sensitivity the case fatality rate of BC patients with M/D breasts will be higher than that of patients with F breasts, and (4) screened women with M/D breasts will experience a higher BC mortality than screened women with F breasts. This study is the first to report on the effect of breast density on BC mortality in screened women.

## Materials and methods

This study was based entirely on data available in administrative registers; it was approved by the Danish Data Inspection Agency. The Copenhagen mammography screening programme started on 1 April 1991 (Vejborg *et al*, 2002). The programme targets women aged 50–69 years at the start of each biennial invitation round. All women are personally invited to participate. About 40 000 women are invited in each invitation round. From the very beginning, all screening has taken place at one clinic dedicated to screening and placed at one of the Copenhagen hospitals. The clinic has three Instrumentarium mammography equipments (Instrumentanium Corp., Imaging Division, Tuusula, Finland) changed over time from Alpha III to Alpha RT. Over time, only two radiologists have been in charge of the screening programme, the first one in 1991–96, and the second (IV) still heads the programme. Attending women are asked to fill in a short questionnaire on hormone therapy (HRT) use, earlier breast surgery, family history of BC, and eventual suspicion of a breast lump. These data were, however, not entered in the database. All screens are taken by the radiographers, and the attending women do not see the radiologist. All mammograms are centrally evaluated independently by two radiologists, to whom only the age of the women and the questionnaire answers are available. At subsequent screens, mammograms are compared with those taken in the earlier screening rounds.

At the first screen, all women had two views taken of each breast, a craniocaudal and an oblique view. During the study period, breast density was dichotomised into F breasts, equivalent to BI-RADS (Breast Imaging Reporting and Data System (BI-RADS) Atlas, 2008) density code 1 and part of code 2, and M/D breasts. Women with a negative screening test and F breasts were scheduled to have only an oblique view made at the next screen, whereas women with a negative screening test and M/D breasts were scheduled for two views. The decision on one or two views was based entirely on density. Density was also evaluated at subsequent screens. The dichotomised density codes were entered in the database for the mammography screening programme and used in this study.

Data from the first five biennial invitation rounds were retrieved from the administrative file of the mammography screening programme. Data on date of death or emigration came from the Central Population Register, on cause of death from the Cause of Death Register, and on incident BC cases from the Cancer Register, the Danish Breast Cancer Cooperative Group, and from the screening data. Incident cases of BC included both invasive BC and DCIS. Data on oestrogen receptor status were collected by the Danish Breast Cancer Cooperative Group from local pathology laboratories, in which primarily immunohistochemical tests were used. A tumour was classified as positive if 10% or more of the cells stained positive. Progesterone receptor status was available only for 37% of invasive cancers, so these data were not included in the analysis. The data files were linked using the unique personal identification numbers issued to all persons with a permanent address in Denmark.

### Analysis

For the analysis of BC incidence, BC mortality and case fatality, women diagnosed before entering the screening programme were excluded, as were women diagnosed with BC at first screen, because they never received a breast density code. For BC incidence, women were followed up from their first negative screen – taking place between 1 April 1991 and 1 April 2001 – until BC diagnosis, death, emigration, or end of follow-up, which was on 1 April 2001. All incident BC cases during the follow-up period were included in the analysis irrespective of whether the mode of detection was at screening, as interval cancer up until 2 years after last screen, or diagnosed more than 2 years after the women left the screening programme. For case fatality, women were followed up from date of diagnosis with BC until date of death, emigration, or end of follow-up on 1 April 2001. For BC mortality, women were followed up from their first negative screen until date of death, emigration, or end of follow-up.

For the sensitivity analysis, screen-detected and interval cancers were defined as above, and the sensitivity of mammography screening in M/D breasts compared with that of F breasts was calculated as the age-adjusted ratio of the odds for interval cancers *versus* screen-detected cancers in the two groups (Mandelson *et al*, 2000; Chiarelli *et al*, 2006).

Person years, incident BC cases, and BC deaths were divided into groups corresponding to the breast density status. At each screen, the woman entered the status recorded for her at that screen, and she remained in this status until end of follow-up unless her status changed at a later screen; if so, she changed status. If a BC was diagnosed at screening, the woman remained for the case fatality and mortality analysis in the status group recorded at her earlier screen until the end of follow-up. BC incidence and mortality were compared between the two breast density groups, and age-adjusted rate ratios were calculated using Poisson regression. The low breast density group was used as the baseline. Poisson regression was used also for comparison of the age-adjusted case fatality rates between BC patients with M/D and F breasts, respectively, in which death from any cause was counted as an event.

## Results

During the first 10 years of the Copenhagen mammography screening programme, a total of 134 640 screens were made and 989 BC cases detected, Table 1. In total, 46% of the negative screens were taken of F breasts. This proportion declined over time from 53% in the first round in 1991–1993 to 36% in the fifth round in 1999–2001. This decline was most pronounced in women aged 50–54 years at the time of screening, in which the percentage changed from 42% in 1991–1993 to 21% in 1999–2001, whereas only a small change was seen over time in the oldest women. The 134 650 screens were taken in a total of 48 052 women, of whom 90% experienced no change in density status during their participation in the programme, 8% experienced one, and the remaining 2% of women experienced two to four shifts.

In the F breast group, in total 158 950 person years were accumulated for the BC mortality analysis, Table 2. The 315 incident BCs, comprised 159 screen-detected, 65 interval, and 91 cancers diagnosed after leaving the screening programme. The interval cancers constituted 29% of the total of screen-detected and interval cancers. In total, 37 BC deaths were observed in the F breast group, resulting in a BC mortality rate of 23 per 100 000 person years. In the M/D breast group, in total 157 594 person years were accumulated. The 694 incident BCs comprised 312 screen-detected, 214 interval, and 168 cancers diagnosed after leaving the screening programme. The interval cancers constituted 41% of the total of screen-detected and interval cancers. In total, 53 BC deaths were observed in the M/D breast group, a BC mortality rate of 34 per 100 000 person years. The age-adjusted odds ratio for an interval cancer *vs* a screen-detected cancer was 1.62 (95% confidence interval, CI, 1.14–2.30) in women with M/D breasts compared with women with F breasts.

The BC incidence rate ratio, as estimated in the Poisson regression using the F breast group as baseline, was 2.45 (95% CI 2.14–2.81), being 2.39 (95% CI 2.08–2.75) for invasive cancer and 3.40 (95% CI 2.02–5.73) for DCIS, Table 3. For invasive cancer, the rate ratios were 2.53 (95% CI 2.13–3.02) for oestrogen receptor-positive cancers and 1.53 (95% CI 1.11–2.12) for oestrogen receptor-negative cancers. The case fatality rate of BCs in M/D breasts was 0.60 (95% CI 0.43–0.84) of that of BCs in F breasts. The age-adjusted BC mortality rate ratio was 1.78 (95% CI 1.17–2.72).

## Discussion

On the basis of the Copenhagen mammography screening programme, we tested four hypotheses on differences between M/D and F breasts. The first was confirmed, as the sensitivity of mammography was lower with M/D than with F breasts indicated by the risk of an interval *vs* a screen-detected cancer being 1.6. The second was also confirmed, as BC incidence was 2.5 times higher in women with M/D than in those with F breasts. The third hypothesis was, however, not confirmed, as the relative risk (RR) for the case fatality rate of cancers in M/D compared with F breasts was 0.6. The fourth hypothesis concerning BC mortality was confirmed, but the better case fatality rate partly compensated for the higher incidence in women with M/D breasts, resulting in BC mortality for these women being only 1.8 times that of women with F breasts.

In this study, women were screened in the age group 50–69 years, and on average, 54% of the women had M/D breasts deemed from the recommendation of two-view mammography at next screen. As our cutoff point for M/D breasts included BI-RADS density codes 4, 3, and part of code 2, this was well in accordance with an earlier study, in which 13% of screens had BI-RADS density code 1; 53% had code 2; and 34% had codes 3 or 4 (Kerlikowske *et al*, 2007). Our data showed a drift over time towards the M/D category, which is probably partly explained by an increasing use of HRT during the study period (Olesen *et al*, 1999; Løkkegaard *et al*, 2007).

As the data were collected from comprehensive, administrative registers, the study was not affected by recall or reporting bias. However, it only covered women attending mammography screening, which in Copenhagen included about 80% of targeted women (von Euler-Chelpin *et al*, 2006). There is, nevertheless, no reason to expect that attending women constituted a biased sample, as women do not *a priori* know their breast density.

The odds ratio, of interval *versus* screen-detected cancer, of 1.62 (95% CI 1.14–2.30) between the two groups, was in line with earlier studies indicating a lower sensitivity of mammography in M/D than in F breasts (Mandelson *et al*, 2000; Chiarelli *et al*, 2006).

The rate ratio of incident BC was 2.45 (95% CI 2.14–2.81) in women with M/D compared with those with F breasts. Taking into account the difference in the cutoff points used for the M/D breast category across studies, our result was well in line with earlier findings (McCormack and dos Santos Silva, 2006; Boyd *et al*, 2007). One could argue that the increased incidence in women with M/D breasts in our study was an artefact because of the more intensive screening using two-view mammography. If this was the case, a greater sensitivity in this group would be expected, which was not so.

Despite the lower sensitivity of mammography screening in M/D than in F breasts, we found that patients with M/D breasts had a better survival, with a case fatality rate ratio of 0.60 (95% CI 0.43–0.84). Using an alternative calculation with competing risks only slightly changed the case fatality rate to 0.56 (95% CI 0.36–0.87). Including only deaths from BC gave a case fatality rate ratio of 0.53 (95%CI 0.34–0.82). An earlier study indicated a slightly worse BC-specific survival for BI-RADS category 2 cancers than for category 1, 3, and 4 cancers (Porter *et al*, 2007).

Although M/D breasts were associated with 2.5 times the risk of BC compared with F breasts, they ended up with a BC mortality of only 1.8 times higher. Data on BC mortality by breast density have not been reported earlier, although hormone use is known to delay breast tissue transition from M/D to F (Persson *et al*, 1997). Current hormone users at recruitment to the Million Women Study had, during a follow-up period of about 4 years, a relative risk of BC incidence of 1.66 (95% CI 1.60–1.72) compared with never users, whereas their relative BC mortality risk was only 1.22 (95%CI 1.05–1.41) (Beral *et al*, 2003). A similar pattern was seen in the Danish Nurses’ Health Study, in which the current HRT use was associated with a rate ratio of 2.42 (95% CI 1.81–3.26) for BC incidence and of 1.97 (95% CI 1.14–3.42) for BC mortality (Stahlberg *et al*, 2005).

The excess BC risk in women with M/D breasts came mainly from oestrogen receptor-positive tumours, RR 2.53 (95% CI 2.13–3.02), although these women also had an excess risk of oestrogen receptor-negative tumours, RR 1.53 (95% CI 1.11–2.12). This accorded with data from an earlier study (Ziv *et al*, 2004). Oestrogen receptor-positive BC patients have a better 5-year survival than oestrogen receptor-negative patients (Bentzon *et al*, 2008), and the oestrogen receptor status data in our study were therefore in line with our finding on case fatality.

Participants in the Copenhagen mammography screening programme on average experienced a 37% lower BC mortality than expected in the absence of screening (Olsen *et al*, 2005). A special effort using two-view mammography was made to diagnose BC in women with M/D breasts, but was, however, not sufficient to bring the BC mortality of screening participants with M/D breasts in line with that of participants with F breasts. This is not surprising, as the sensitivity despite the special effort was lower in women with M/D breasts than in women with F breasts.

This study has shown that, although BC is more frequent in women with M/D than in those with F breast, the BCs of women with M/D breasts seem on average to be less severe.

## Figures and Tables

**Table 1:** Participants in first–fifth invitation round of the mammography screening programme in Copenhagen, Denmark, 1991–2001. Women aged 50–69 years at the start of each invitation round

	**Outcome of screening**							
	**Breast cancer**				**Negative,**			**One view in %**
**Invitation round**	**Invasive**	**DCIS**	**Total**	**Negative, fatty**	**mixed/dense**	**Others^a^**	**Total**	**of all negative**
First, 1 April 1991–25 April 1993	316	44	360	15 805	14 197	33	30 395	53
Second, 26 April 1993–31 May 1995^b^	162	21	183	14 615	13 779	28	28 605	51
Third, 1 June 1995–24 March 1997	136	18	154	11 666	13 317	13	25 150	47
Fourth, 25 March 1997–19 April 1999	129	18	147	10 670	14 602	9	25 428	42
Fifth, 20 April 1999–31 March 2001	123	22	145	8985	15 928	4	25 062	36
Total	866	123	989	61 741	71 823	87	134 640	46

Abbreviation: DCIS=ductal carcinoma *in situ.*

a Coding errors.

b The invitation round targeted women aged 50–71 years.

**Table 2:** Number of person years at risk, screen-detected breast cancers, interval breast cancers, other breast cancers, and breast cancer deaths by density code and age at diagnosis

	**Fatty breasts**						**Mixed/dense breasts**					
		**Breast cancer cases^a^**						**Breast cancer cases^a^**				
**Age (years)**	**Person years breast cancer mortality**	**S^b^**	**I^c^**	**O^d^**	**Total**	**Breast cancer deaths**	**Person years breast cancer mortality**	**S^b^**	**I^c^**	**O^d^**	**Total**	**Breast cancer deaths**
50–54	14 811	5	4	0	9	0	31 435	33	39	8	80	3
55–59	30 621	24	11	8	43	3	40 464	88	56	22	166	7
60–64	33 710	55	15	13	83	6	30 541	80	52	36	168	14
65–69	37 236	53	18	12	83	12	27 242	86	38	18	142	12
70–74	32 072	22	17	27	66	11	21 347	25	29	55	109	9
75–79	10 500	0	0	31	31	5	6565	0	0	29	29	8
Total	158 950	159^e^	65^e^	91	315	37	157 594	312^e^	214^e^	168	694	53

Abbreviation: CI=confidence intervals; DCIS=ductal carcinoma *in situ*.

a Invasive breast cancer and DCIS.

b Screen-detected breast cancer.

c Breast cancer diagnosed <2 years after the last screening date.

d Breast cancer diagnosed ⩾2 years after the last screening date.

e Age-adjusted odds ratio of interval cancer *versus* screen-detected cancer in women with two-view compared with women with one-view mammography 1.62 (95% CI 1.14–2.30).

**Table 3:** Breast cancer incidence by stage and oestrogen-receptor status, breast cancer case fatality and breast cancer mortality by density

	**Fatty breasts**		**Mixed/dense**			
	**Number of cases**	**Person years**	**Number of cases**	**Person years**	**Rate ratio^a^**	**95% confidence interval**
*Breast cancer incidence*						
Total	315	158 017	694	155 110	2.45	2.14–2.81
Invasive only	296	158 017	634	155 110	2.39	2.08–2.75
DCIS only	19	158 017	60	155 110	3.40	2.02–5.73
						
*Invasive breast cancer incidence*						
Oestrogen receptor positive	188	158 017	421	155 110	2.53	2.13–3.02
Oestrogen receptor negative	64	158 017	94	155 110	1.53	1.11–2.12
Oestrogen receptor unknown	44	158 017	119	155 110	3.05	2.15–4.33
Breast cancer case fatality	60^b^	1988	86	4725	0.60	0.43–0.84
Breast cancer mortality	37^c^	158 950	53	157 594	1.78	1.17–2.72

Abbreviation: DCIS=ductal carcinoma *in situ.*

a Age-adjusted, Poisson regression.

b All deaths.

c Breast cancer deaths.

## References

[bib1] Bentzon N, Düring M, Rasmussen BB, Mouridsen H, Kroman N (2008) Prognostic effect of estrogen receptor status across age in primary breast cancer. Int J Cancer 122: 1089–1094 17960621 10.1002/ijc.22892

[bib2] Beral V, Million Women Study Collaborators (2003) Breast cancer and hormone-replacement therapy in the Million Women Study. Lancet 362: 419–427 12927427 10.1016/s0140-6736(03)14065-2

[bib3] Boyd NF, Guo H, Martin LJ, Sun L, Stone J, Fishell E, Jong RA, Hislop G, Chiarelli A, Minkin S, Yaffe MJ (2007) Mammographic density and the risk and detection of breast cancer. N Engl J Med 356: 227–236 17229950 10.1056/NEJMoa062790

[bib4] Breast Imaging Reporting and Data System (BI-RADS) Atlas (2008) Accessed 18 December 2007 on: http://www.acr.org/SecondaryMainMenuCategories/ACRStore/FeaturedCategories/QualityandSafety/birads_atlas.aspx

[bib5] Chiarelli AM, Kirsh VA, Klar NS, Shumak R, Jong R, Fishell E, Yaffe MJ, Boyd NF (2006) Influence of patterns of hormone replacement therapy use and mammographic density on breast cancer detection. Cancer Epidemiol Biomarkers Prev 15: 1856–1862 17035392 10.1158/1055-9965.EPI-06-0290

[bib6] Ciatto S, Visioli C, Paci E, Zappa M (2004) Breast density as a determinant of interval cancer at mammographic screening. Br J Cancer 90: 393–396 14735182 10.1038/sj.bjc.6601548 PMC2409545

[bib7] Kerlikowske K, Ichikawa L, Miglioretti DL, Buist DS, Vacek PM, Smith-Bindman R, Yankaskas B, Carney PA, Ballard-Barbash R, National Institute of Health Breast Cancer Surveillance Consortium (2007) Longitudinal measurement of clinical mammographic breast density to improve estimation of breast cancer risk. J Natl Cancer Inst 99: 386–395 17341730 10.1093/jnci/djk066

[bib8] Løkkegaard E, Lidegaard O, Møller LN, Agger C, Andreasen AH, Jørgensen T (2007) Hormone replacement therapy in Denmark, 1995–2004. Acta Obstet Gynecol Scand 86: 1342–1351 17963062 10.1080/00016340701505523

[bib9] Mandelson MT, Oestreicher N, Porter PL, White D, Finder CA, Taplin SH, White E (2000) Breast density as a predictor of mammographic detection: comparison of interval- and screen-detected cancers. J Natl Cancer Inst 92: 1081–1087 10880551 10.1093/jnci/92.13.1081

[bib10] McCormack VA, dos Santos Silva I (2006) Breast density and parenchymal patterns as markers of breast cancer risk: a meta-analysis. Cancer Epidemiol Biomarkers Prev 15: 1159–1169 16775176 10.1158/1055-9965.EPI-06-0034

[bib11] Olesen C, Steffensen FH, Sørensen HT, Nielsen GL, Olsen J, Bergman U (1999) Low use of long-term hormone replacement therapy in Denmark. Br J Clin Pharmacol 47: 323–328 10215757 10.1046/j.1365-2125.1999.00879.x PMC2014221

[bib12] Olsen AH, Njor SH, Vejborg I, Schwartz W, Dalgaard P, Jensen MB, Tange UB, Blichert-Toft M, Rank F, Mouridsen H, Lynge E (2005) Breast cancer mortality in Copenhagen after introduction of mammography screening: cohort study. BMJ 330: 220 15649904 10.1136/bmj.38313.639236.82 PMC546064

[bib13] Persson I, Thurfjell E, Holmberg L (1997) Effect of estrogen and estrogen-progestin replacement regimens on mammographic breast parenchymal density. J Clin Oncol 15: 3201–3207 9336356 10.1200/JCO.1997.15.10.3201

[bib14] Porter GJ, Evans AJ, Cornford EJ, Burrell HC, James JJ, Lee AH, Chakrabarti J (2007) Influence of mammographic parenchymal pattern in screening-detected and interval invasive breast cancers on pathologic features, mammographic features, and patient survival. Am J Roentgenol 188: 676–683 17312053 10.2214/AJR.05.1950

[bib15] Stahlberg C, Lynge E, Andersen ZJ, Keiding N, Ottesen B, Rank F, Hundrup YA, Obel EB, Pedersen AT (2005) Breast cancer incidence, case-fatality and breast cancer mortality in Danish women using hormone replacement therapy – a prospective observational study. Int J Epidemiol 34: 931–935 15914504 10.1093/ije/dyi103

[bib16] Vejborg I, Olsen AH, Jensen MB, Rank F, Tange UB, Lynge E (2002) Early outcome of mammography screening in Copenhagen 1991–99. J Med Screen 9: 115–119 12370322 10.1136/jms.9.3.115

[bib17] von Euler-Chelpin M, Olsen AH, Njor S, Vejborg I, Schwartz W, Lynge E (2006) Women's patterns of participation in mammography screening in Denmark. Eur J Epidemiol 21: 203–209 16547835 10.1007/s10654-006-0002-1

[bib18] Ziv E, Tice J, Smith-Bindman R, Shepherd J, Cummings S, Kerlikowske K (2004) Mammographic density and estrogen receptor status of breast cancer. Cancer Epidemiol Biomarkers Prev 13: 2090–2095 15598766

